# Acute promyelocytic leukemia presenting with atypical basophils

**DOI:** 10.1002/ccr3.2686

**Published:** 2020-02-05

**Authors:** Afshin Shameli, Kareem Jamani

**Affiliations:** ^1^ Division of Hematology Alberta Precision Laboratories, and Department of Pathology and Laboratory Medicine University of Calgary Calgary AB Canada; ^2^ Division of Hematology and Hematological Malignancies Department of Medicine University of Calgary Calgary AB Canada

**Keywords:** acute promyelocytic leukemia, basophils, flow cytometry, *PML‐RARA*

## Abstract

We describe a case of acute promyelocytic leukemia with circulating aberrant basophils. Recent studies have shown that basophilic differentiation is not uncommon in APL and likely under‐recognized in morphologic and immunophenotypic assessments.

## Graphical abstract

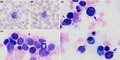

## 1 INTRODUCTION

**Figure 1:**
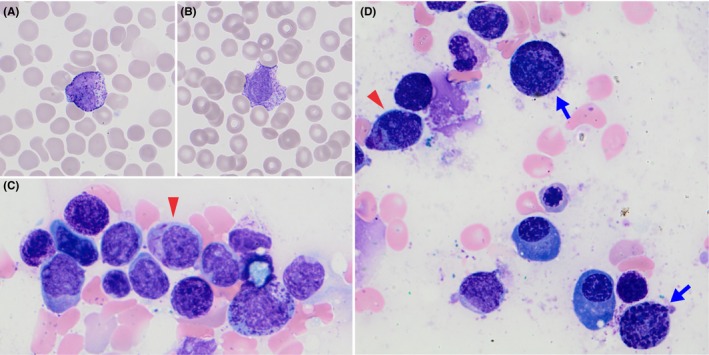
A‐B, Peripheral blood smear (1000× magnification) displaying two atypical basophils with abnormal granulation. C‐D, Bone marrow aspirate smear (1000x magnification) showing promyelocytes/blasts with Auer rods (red arrowheads), other promyelocytes/blasts with or without cytoplasmic granulation, and immature and atypical basophils with abnormally coarse cytoplasmic granulation (blue arrows)

A 63‐year‐old woman presented to the emergency room with leg swelling and dyspnea and was diagnosed with deep venous thrombosis and bilateral pulmonary emboli. Complete blood count (CBC) revealed mild thrombocytopenia and neutropenia. She was treated with apixaban. A repeat CBC 1 month later revealed progressive thrombocytopenia (109 × 10^9^/L) and neutropenia (0.5 × 10^9^/L). Peripheral blood smear showed relatively increased atypical basophils (15%, 0.2 × 10^9^/L) with hypogranulation/fine granulation (Figure 1, panels A‐B). Bone marrow assessment revealed 22% basophilic elements and 28% abnormal promyelocytes/blasts some with several Auer rods (Figure 1, panels C‐D). Flow cytometry confirmed distinct populations of promyelocytes/blasts (21%, CD45^dim^ CD34^‒^ CD117^++^ CD33^++^ CD13^+/‒^ HLA‐Dr^‒^ CD11b^‒^ CD123^‒/+^ CD7^‒^) and basophils (15%, CD45^mod^ CD34^‒^ CD117^+/‒^ CD33^++^ CD13^++^ HLA‐Dr^‒^ CD11b^++^ CD123^+^ CD7^+/‒^). Fluorescent in situ hybridization showed t(15;17)(q22;q12) in 44.5% of nuclei, suggesting that both subsets carry the translocation. Acute promyelocytic leukemia with *PML‐RARA* (APL) was diagnosed.

Basophilic differentiation of APL at presentation or after therapy is rarely reported.^1^ A recent study showed that basophilic traits of APL promyelocytes/blasts happen in up to one‐third of patients and are associated with increased risk of bleeding and worse survival,^2^ suggesting that such traits are under‐recognized by cytomorphology and flow cytometry.

## CONFLICT OF INTEREST

Nothing to report.

## AUTHOR CONTRIBUTIONS

AS: wrote the first draft of the paper, prepared the figure, and reviewed literature. KJ: revised the paper, included clinical information, and obtained patient consent.
